# Rapid authentication of endangered *Cistanche* Herba (*Rou Cong Rong*) using a high-throughput multi-SNP panel and MALDI-TOF MS platform

**DOI:** 10.3389/fpls.2025.1677826

**Published:** 2026-01-26

**Authors:** Rong Lei, Yixia Cao, Yifen Yang, Haolong Cong, Limei Li, Xin Li, Junxia Shi

**Affiliations:** 1 Chinese Academy of Quality and Inspection & Testing, Beijing, China; 2 China Certification & Inspection Group, Beijing, China; 3 Technology Center of Chengdu Customs District, Chengdu, Sichuan, China; 4 Jilin Provincial Academy of Forestry Science, Changchun, China; 5 Technology Center of Dalian Customs District, Dalian, China

**Keywords:** *C. deserticola*, Cistanche Herba, MALDI-TOF MS, multiplex PCR, SNP

## Abstract

*Cistanche* Herba (*Rou Cong Rong*), a critically endangered edible tonic and medicinal plant, is traditionally valued for its nephroprotective and kidney-yang tonifying properties. However, wild populations are declining due to habitat loss, overharvesting, and increasing market demand, leading to widespread adulteration in commercial supplies. Conventional authentication methods, such as morphological examination, photochemical profiling, and ITS/ITS2 barcoding, often fail with processed materials due to DNA degradation. To overcome these limitations, we developed a high-throughput single-nucleotide polymorphism (SNP) genotyping platform that integrates multiplex PCR with MALDI-TOF mass spectrometry, targeting validated nuclear ITS and chloroplast-encoded ribosomal protein large subunit 16 (*rpl16*) loci. The assay utilizes four diagnostic SNPs specific to *C. deserticola*, allowing unambiguous differentiation from six adulterants. It demonstrates high sensitivity, detecting 0.07% genomic DNA (6.8 pg/μL) in mixed samples and 1% *C. deserticola* powder in dried tissue mixture. When validated on 27 dried specimens, the method showed 100% concordance with Sanger sequencing while reducing the total analysis time to approximately 10 hours. By overcoming the resolution limitations of traditional techniques, this approach provides a rapid and scalable solution to combat herbal substitution, support CITES compliance, ensure the integrity of functional foods and traditional medicines.

## Introduction

*Cistanche deserticola* Y. C. Ma (commonly known as “*Rou Cong Rong*” in Chinese), traditionally regarded as a “geo-authentic medicinal herb” and esteemed as “Desert *Cistanche*” for its exceptional tonic (Zhu et al., 2025) and medicinal properties (Wang et al., 2012). The stems of *C. deserticola* are rich in bioactive compounds such as phenylethanoid glycosides, and iridoids, which contribute to a broad spectrum of pharmacological activities, including immunomodulatory, androgen-like, antioxidative, anti-apoptotic, anti-nociceptive, anti-inflammatory, anti-fatigue, anti-aging, laxative, and neuroprotective activities (Wang et al., 2017). In contrast, *Cistanche tubulosa* (Schrenk), is scarcely documented in ancient texts of Traditional Chinese Medicine and has only gained widespread use in modern times. Although included in the Chinese Pharmacopoeia (Chinese Pharmacopoeia, 2020), it has traditionally been considered as a substitute for *C. deserticola*, with less historical recognition of its geo-authenticity and efficacy. *Cistanche deserticola* generally contained higher concentrations of echinacoside and acteoside, which underlies its superior pharmacological potency and higher market price than *C. tubulosa*. Owing to its stronger traditional reputation, generally greater abundance of active compounds, and scarcer wild resources, *C. deserticola* commands a significantly higher market price than *C. tubulosa.* Therefore, accurate species differentiation is essential to ensure therapeutic efficacy, protect consumer rights, and guarantee the safety and quality of herbal products.

*Cistanche deserticola* and *C. tubulosa* are obligate parasitic plants endemic to the arid regions of northwestern China. However, due to their narrow habitat specificity, limited geographic distribution, and overharvesting for medicinal use, *C. deserticola* has been classified as endangered in the *Chinese Angiosperm Red List*, with international trade regulated under CITES (Convention on International Trade in Endangered Species) (https://cites.org/eng). The scarcity of wild resources has prompted the proliferation of adulterants in commercial markets, including *Cynomorium songaricum* Rupr. (“Suo Yang”), *Cistanche sinensis* Beck, *Cistanche salsa* (C. A. Meyer) Beck, *Orobanche pycnostachya* Hance, *Orobanche coerulescens* Stephan, *Boschniakia rossica* (Cham. et Schlecht.) and other *Cistanche* species (Sun et al., 2012), posing significant risks to drug safety and efficacy. Conventional identification methods are fraught with limitations: microscopic analysis lacks specificity, especially for processed materials (Wang et al., 2018), while chromatographic profiling of bioactive components using high performance chromatography (HPLC)-based methods cannot reliably distinguish adulterants like *C. salsa* (Chen et al., 2007; Li et al., 2001) and *C. sinensis* (Liu et al., 2013) due to overlapping phytochemical profiles (Jiang and Tu, 2009).

Molecular approaches utilizing DNA sequence data have emerged as reliable tools for species authentication in herbal medicine. The use of standardized DNA markers facilitate accurate identification of plant species in herbal products (de Boer et al., 2015; Hebert et al., 2003; Kress et al., 2005). Phylogenetic studies of the ribosomal DNA internal transcribed spacer 2 (ITS2) region using neighbor-joining (NJ) and Kimura 2-parameter (K2P) methods, indicated that *C. deserticola* (229 bp) and *C. tubulosa* (233 bp) are most closely related to *C. salsa* (Sun et al., 2012). However, the applicability of ITS2-based identification is compromised in processed materials like Chinese patent medicines, where DNA degradation occurs (Newmaster et al., 2013). To address this, species-specific “mini-barcodes” (100–200 bp) (Dubey et al., 2011; Hajibabaei et al., 2006; Lo et al., 2015; Meusnier et al., 2008) and even shorter nucleotide signatures (<100 bp) have been developed and successfully applied to detect adulterants in herbal formulations, such as *Lonicerae japonicae* Flos (Gao et al., 2017), Ginseng (Liu et al., 2016), *Angelicae Sinensis* Radix (Wang et al., 2016), *Pinelliae Rhizoma* (Zhang et al., 2022) and *Cistanche* Herba (Wang et al., 2018). A notable limitation of these sequence-dependent methods is their requirement for sequencing and computational analysis, which hinders broad adoption in resource-constrained laboratories.

The MassARRAY platform integrates multiplex PCR amplification with matrix-assisted laser desorption ionization time-of-flight mass spectrometry (MALDI-TOF MS), enabling highly sensitive and simultaneous detection of multiple DNA targets (Ellis and Ong, 2017). Over the past decade, its versatility has been demonstrated across diverse molecular diagnostics, from the detection of cancer-related mutations (Min et al., 2016; Shah et al., 2020; Svidnicki et al., 2015; Verma et al., 2020; Wang et al., 2019) and susceptibility variants to infectious agents including *Mycobacterium tuberculosis* (Yang et al., 2023), bacterial pathogens (Croxatto et al., 2012), enteroviruses (Peng et al., 2013), respiratory pathogens (Zhao et al., 2021), and SARS-CoV-2 subtyping (Almutawa et al., 2023; Hernandez et al., 2021; Rybicka et al., 2021; Wacharapluesadee et al., 2023). The workflow comprises multiplex PCR amplification of genomic regions, single-base probe extension to generate mass-distinct products, and subsequent MALDI-TOF MS analysis on pre-spotted assay chips (Ellis and Ong, 2017).

This study aims to design and validate a mass spectrometry-based DNA assay for the rapid, accurate and high-throughput authentication of *C. deserticola*, and to differentiate it from common substitute *C. tubulosa*, as well as from other adulterant species. By targeting conserved regions within the ITS genes and the chloroplast-encoded ribosomal protein large subunit 16 (*rpl16*) loci (Tomari et al., 2002), we identified multiple single nucleotide polymorphisms (mSNPs) capable of differentiating *C. deserticola* and *C. tubulosa* from related species and counterfeit materials. This approach provides a precise diagnostic tool for TCM quality control while overcoming the technical limitations of conventional molecular methods.

## Materials and methods

### Materials and reagents

A plant DNA extraction kit with magnetic beads (Cat# DP 342), Super HiFi PCR mix (Cat# KT213) and molecular-grade nuclease-free water were purchased from Tiangen Biotech Co. Ltd. (Beijing, China). PCR Reagents Set (10×PCR buffer, 25 mM MgCl_2_, 25 mM dNTP mix) (Cat# 21327M) and iPlex Pro Regent Set (10×iPlex Buffer Plus, iPlex Termination Mix, iPlex Enzyme, SAP Buffer, SAP enzyme) (Cat# 10212), SpectroCHIP™ CPM Kit G96 & Resin Kit (Cat# 10600F) were purchased from Agena Bioscience. The Mixer Mill AM 100 was purchased from Ants Scientific Instruments (Beijing, China). The Qubit^®^2.0 Fluorometer and the Qubit^®^dsDNA High Sensitivity (HS) Assay kit (Cat# Q32851) were purchased from Invitrogen (Life technologies, Carlsbad, CA). A Milli-Q water purification system was obtained from Millipore Corp. (Merck KGaA, Darmstadt, Germany). The ddH_2_O obtained from a Milli-Q water purification system was autoclaved at 120 °C for 20 min.

### Sample collections and nucleic acid extraction

Dried plant samples morphically similar to *Cistanche* originating from five transcontinental regions (Egypt, Ethiopia, Kazakhstan, United Arab Emirates and Uzbekistan) were taxonomically validated through a tripartite molecular identification procedures: (1) PCR amplification using *C. deserticola*-specific primers (Cd-F: CGCGCATGGTGGATTCACAATCC; Cd-R: GTTATGCATGAACGTAATGCTC) or *Cistanche* genus-specific primers (Cis-F: CGGTAAATATGCTCTTCAAGC; Cis-R: GTCTATTCTTTCAATGCAAAGG); (2) Bidirectional Sanger sequencing of the resulting amplicons, and (3) phylogenetic confirmation via BLASTn alignment against the NCBI Nucleotide database.

Dried plant tissues were cryogenically pulverized into a fine powder using a Mixer Mill AM 100 with beads in liquid nitrogen. Subsequently, 500 mg of the homogenized powder was transferred into pre-chilled 1.5mL DNase-free microcentrifuge tubes. Genomic DNA was extracted using a magnetic bead-based plant genomic DNA extraction kit (Cat# DP342) in accordance with the manufactures’ protocol. The paramagnetic bead-DNA complexes were washed twice with freshly prepared 80% ethanol, and the purified DNA was eluted in Tris-EDTA buffer. DNA concentration was quantified using the Qubit^®^2.0 system with the dsDNA Qubit^®^dsDNA HS Assay kit as directed by the manufacturer.

### Multitargets design strategy for *Cistanche* species identification using ITS and chloroplast gene

**Figure 1:**
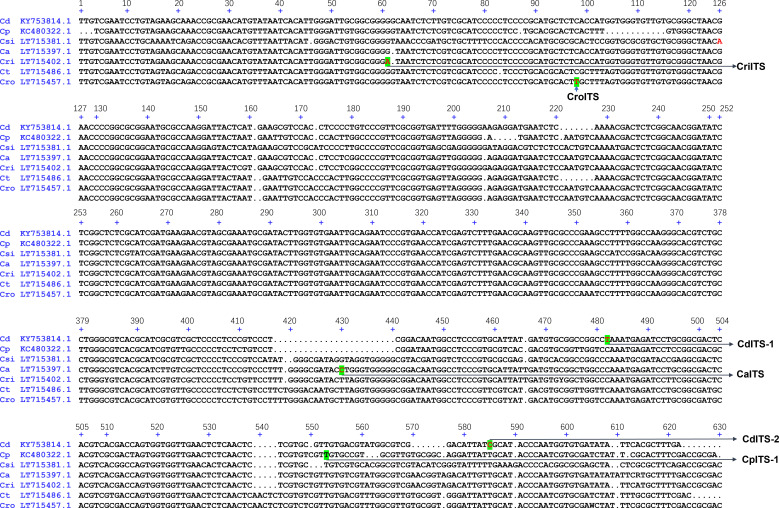
Sequence alignment of ITS genes of *C. deserticola* (Cd), *C. phelypaea* (Cp), *C. sinensis* (Csi), *C. ambigua* (Ca), *C. ridgewayana* (Cri) and *C. rosea* (Cro).

**Figure 2:**
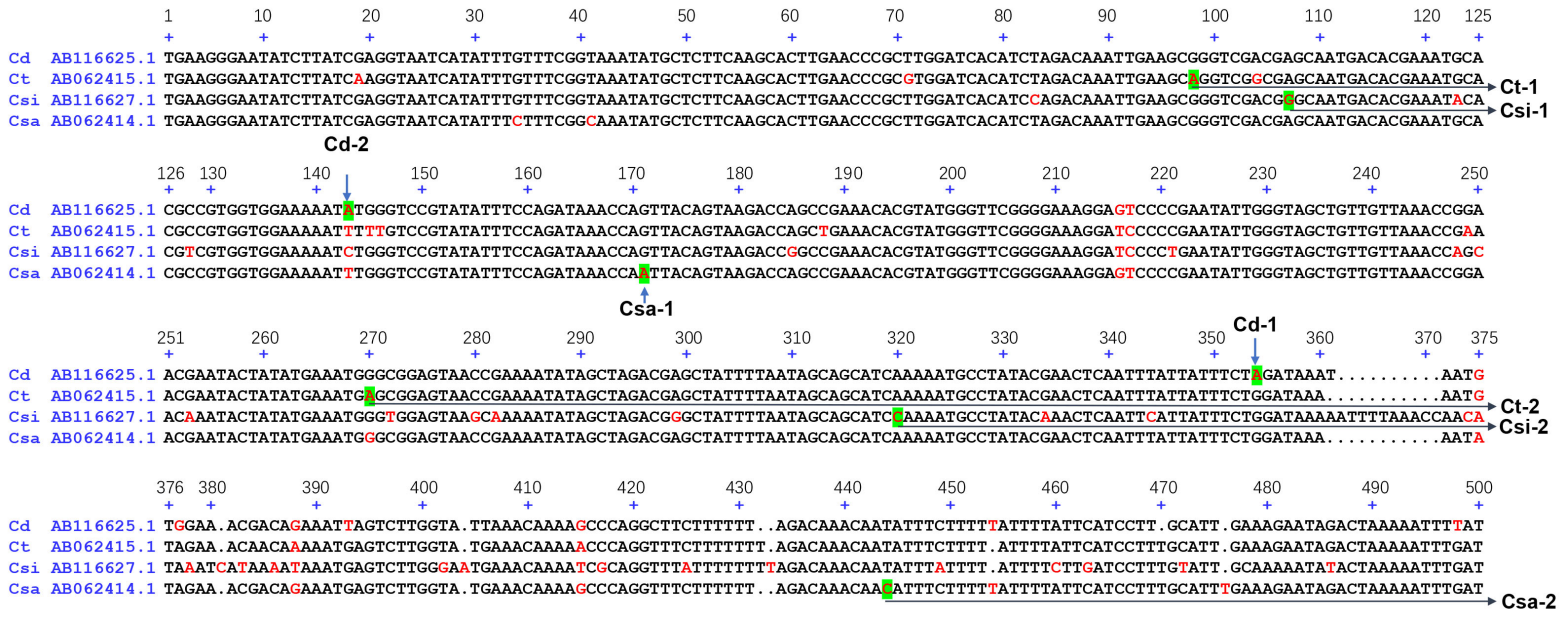
Sequence alignment of *rpl16* genes of *C. deserticola* (Cd), *C. tubulosa* (Ct), *C. salsa* (Csa), *C. sinensis* (Csi).

To overcome the taxonomic limitations within the *Cistanche* genus, we established a dual-locus molecular identification system based on the nuclear ribosomal internal transcribed spacer (ITS) and the chloroplast-encoded ribosomal protein large subunit 16 (*rpl16*) gene. Reference sequences were retrieved from NCBI GenBank, covering six phylogenetically relevant specie for the ITS region, i.e. *C. deserticola* (KY753814.1), *C. phelypaea* (KC480322.1), *C. sinensis* (LT715381.1), *C. ambigua* (LT715397.1), *C. ridgewayana* (LT715402.1), and *C. rosea* (LT715457.1) (**Figure 1**) and four species for rpl16 gene, i.e. *C. deserticola* (AB116625.1), *C. tubulosa* (AB062415.1), *C. salsa* (AB062414.1), *C. sinensis* (AB116627.1) (**Figure 2**). Sequence alignment revealed high interspecific homology (> 89.3%) at both loci, which hindered the design of conventional species-specific PCR primer.

To overcome this constraint, we developed a MassARRAY compatible single nucleotide polymorphism (SNP) genotyping panel targeting diagnostic SNP clusters within hypervariable ITS regions and species-discriminating indels in *rpl16* intronic domains. Primers were designed using the Agena Bioscience ASSAY DESIGN SUITE v2.2 under the following parameters: amplicon length 80-200 bp, PCR primer Tm optimized to 60 °C, incorporation of a 10-mer tag at the 5’-end of PCR primer to ensure a primer mass >8500 Da, extension probe hybridization Tm between 45-90 °C, and extension probe length between 17-28 bp.

To maintain detection resolution via MALDI-TOF MS, the PCR primers and extension probes were allocated to two reaction wells: Well 1 contained Ct-2, Csi-2, CpITS, CaITS, CroITS, CdITS-2, Csa-1, Csa-2 and Csi-1; Well 2 contained Cd-1, Cd-2, CriITS, CdITS-1 and Ct-1. All final PCR primers and extension probe (**Tables 1**, **2**) were synthesized by Sangon Biotech (Shanghai, China).

**Table 1:** PCR primers information.

Tube	Target name	PCR Primers (5’-3’)	Mol. mass (Da)	GC%	Tm (°C)	Amplicon size (bp)
1	Ct-2	ACGTTGGATGTCGGTAGCTGTTGTTAAACC	9268.1	46.7	73.5	112
		ACGTTGGATG ATTGAGTTCGTATAGGCAT	9002.9	41.4	71.1	
	Csi-2	ACGTTGGATGATGGGTGGAGTAAGCAAAA	9095	44.8	74.9	144
		ACGTTGGATGTGTTTCATTCCCAAGACTC	8858.8	44.8	73.5	
	CpITS	ACGTTGGATGAGATGAACTCTCAACTCTCG	9206.1	46.7	73.3	92
		ACGTTGGATGGAATAGATCGCACGATTGGG	9351.2	50	78.5	
	CaITS	ACGTTGGATGTACACGTCACGCATCTTGT	8883.8	48.3	75.6	85
		ACGTTGGATGTATCAATAATGCACGGGAGG	9335.2	46.7	75.5	
	CroITS	ACGTTGGATGACGTAATCTCTCGTCGCATC	9173	50	76.4	66
		ACGTTGGATGTTCGTTAGCCCACACAACAC	9151	50	76.9	
	CdITS-2	ACGTTGGATGGTATGGCGTCGAATGGTAG	9069	51.7	76.6	83
		ACGTTGGATGACTAAACTCAGCGGGTAGTC	9271.1	50	73.3	
	Csa-1	ACGTTGGATGGGTGGAAAAATTTGGGTCCG	9382.2	50	79.9	75
		ACGTTGGATGCCGAACCCATACGTGTTTC	8868.8	51.7	78.1	
	Csa-2	ACGTTGGATGTATAGAAACGACAGAAATGAG	9665.4	38.7	69.8	118
		ACGTTGGATGCTTTCAAATGCAAAGGATG	8980.9	41.4	75.2	
	Csi-1	ACGTTGGATGCTCTTCAAGCACTTGAACCC	9142	50	77.1	88
		ACGTTGGATGTCCACCACGACGTGTATTTC	9173	50	76.9	
2	Cd-2	ACGTTGGATGGTCGCTTGGATCACATCTA	8923.9	48.3	76.2	120
		ACGTTGGATGGGCTGGTCTTACTGTAACTG	9284.1	50	73.9	
	Cd-1	ACGTTGGATGCTATGCCTATACGAACTCAA	9190.1	43.3	72.4	77
		ACGTTGGATGCCAAGACTAATTTCTGTCG	8907.9	44.8	73.2	
	CriITS	ACGTTGGATGTCGCGAACATGTATAATCAC	9230.1	43.3	73.6	141
		ACGTTGGATGCTTCACGAGTAATCCTTGGC	9213	50	76.7	
	CdITS-1	ACGTTGGATGCCTCCCGTGCATTATTGATG	9204	50	79.2	87
		ACGTTGGATGGAGTTGAGAGTTCAACCACC	9271.1	50	75.9	
	Ct-1	ACGTTGGATGACGTGGATCACATCTAGACA	9255.1	46.7	74.9	54
		ACGTTGGATGCCATTTCGTGTCATTGCTCG	9195	50	80.4	

**Table 2:** Extension probe sequence, designed SNP and corresponding molecular mass.

Tube	Target name	UEP Mass (Da)	Extension probe sequence (5’-3’)	SNP	EP prod. mass (Da)	Con. (μM)
1	Ct-2	5128.3	ATTTTCGGTTACTCCGC	A	5455.4	6.92
	Csi-2	5222.4	GTTTGTATAGGCATTTT	C	5509.6	7.09
	CpITS	5424.5	CCGCACAACGCACGGCAC	T	5965.7	7.46
	CaITS	5796.8	CGTCCCTTTGGGGCGATAC	C	6043.9	8.09
	CroITS	5993.9	CACACAACACCCACTAAAGC	T	6265.1	8.41
	CdITS-2	6173	CGTCGAATGGTAGACATTGT	C	6420.2	8.69
	Csa-1	6374.2	CGTATATTTCCAGATAAACCA	A	6645.4	9.00
	Csa-2	7326.8	CAGGTTTCTTTTTTAGACAAACAA	C	7574	10.33
	Csi-1	7929.1	CCACGACGTGTATTTCGTGTCATTGC	G	8176.3	11.09
2	Cd-2	5213.4	GGAAATATACGGACCCA	A	5540.5	7.08
	Cd-1	5414.6	ACTCAATTTATTATTTCT	A	5685.8	7.44
	CriITS	5613.7	GGATGCGACGAGAGATTA	A	5940.8	7.78
	CdITS-1	5740.7	TCGCCGCAGGATCCCATTT	T	6011.9	8.00
	Ct-1	5805.8	CATCTAGACAAATTGAAGC	A	6077	8.11

UEP, unextended probe; EP prod, extended product.

### Multiplex SNP profiling on the MassARRAY platform

Multiplex PCR primer pools were prepared by combining equimolar aliquots of individual oligonucleotide working solutions (100 μM stock), followed by dilution with RNase/DNase-free water to a final concentration of 1 μM per primer pair. Extension probe mixtures were prepared by stoichiometrically blending of unextended probes (UEPs, 500 μM stock) to achieve species-specific concentration gradients as specified in **Table 2**.

PCR amplification was conducted using the PCR Reagent Kit (Cat# 21327M) in 5 μL reaction volumes containing 3 μL of master mix (comprising 1 μL of 1μM forward/reverse primers, 0.4 μL of 25 mM MgCl_2_, 0.1 μL of 25 μM dNTP, 0.5 μL of PCR buffer, and 0.2 μL of 50U/μL Taq enzyme), and 2 μL of template DNA. Thermal cycling conditions consisted of initial denaturation at 95 °C for 2 min; 45 cycles of 95 °C for 30 s, 56 °C for 30 s, and 72 °C for 1 min; followed by a final extension at 72 °C for 5 min.

After amplification, unincorporated dNTPs were enzymatically degraded by adding 2 μL of shrimp alkaline phosphate (SAP) mixture (1.56 μL of RNase/DNase-free water, 0.17 μL of 10 × SAP Buffer, 0.3 μL of SAP enzyme) (Cat# 10212) to the PCR products, followed by incubation at 37 °C for 40 min and enzyme inactivation at 85 °C for 5 min.

Single-base extension was performed using iPLEX Pro Reagent Set (Cat# 10212) in a 9 μL volume containing 7 μL of SAP-treated amplicons, 2 μL of extension master mix (0.62 μL of HPLC-grade water, 0.2 μL of 10 × iPLEX Buffer Plus, 0.2 μL of iPLEX Termination mix, 0.94 μL of extension primer, 0.04 μL of 33 U/μL iPlex Pro Enzyme). The extension program included an initial step at 95 °C for 30 s, followed by 40 cycles of 95 °C for 5 s, and an inner loop of 5 cycles at 52 °C for 5 s and 80 °C for 5 s, with a final extension at 72 °C for 3 min.

Reaction products were desalted using SpectroCLEAN resin and centrifuged at 3000 g for 5 min to remove ionic interference prior to mass spectrometry analysis. A total of each purified extension products (15 nL) were automatically dispensed into a 96-spot SpectroCHIP (Agena Bioscience). Mass spectra were acquired in real-time using the MassARRAY RT v4.1 software with adaptive baseline corrections. Nuclease-free water was incorporated as blank control per each 96-well plate to monitor potential environmental contamination.

### Analytical validation of *Cistanche* Herba SNP genotyping platform

The discriminatory capacity of the MassARRAY platform was assessed using genomic DNA from authenticated *C. deserticola and C. tubulosa*. To evaluate the detection sensitivity for *C. deserticola*, artificial mixtures containing varying concentrations of *C. deserticola* and *C. tubulosa* genomic DNA were prepared. Specifically, *C. deserticola* DNA (quantified as 6.8 ng/μL) was serially10-fold diluted to concentrations of 6.8 ng/μL, 0.68 ng/μL and 6.8 pg/μL. Each dilution was mixed with 9.4 ng/μL *C. tubulosa* genomic DNA at 1:1 (v/v) ratio. These prepared mixtures were subsequently analyzed with the MassARRAY assay.

### Field deployment for species authentication

The diagnostic performance of the optimized MassARRAY assay was further validated using both authenticated adulterated commercial samples and intercepted suspicious *Cistanche* materials. For the preparation of authenticated adulterated samples, dried tissues of *C. deserticola* and *C. tubulosa* were cryogenically ground into a fine powder using a Mixer Mill AM 100 with beads in liquid nitrogen. Different quantities of *C. deserticola* powder (20 mg, 200 mg and 400 mg) were mixed with corresponding amounts of *C. tubulosa* powder (1980 mg, 1800 mg and 1600 mg), respectively, to obtain homogenized tissue powder mixture. Genomic DNA was then extracted from these samples using a plant genomic DNA extraction kit, and analyzed with the MassARRAY assay.

In addition, 27 cross-border samples originating from five transcontinental regions (Egypt, Ethiopia, Kazakhstan, United Arab Emirates and Uzbekistan) were subjected to species identification using the MassARRAY platform. The results were compared with Sanger sequencing and BLASTn alignment against the NCBI Nucleotide database for verification.

## Results

### Strategy for pharmacopeial authentication of *Cistanche* species

**Figure 3:**
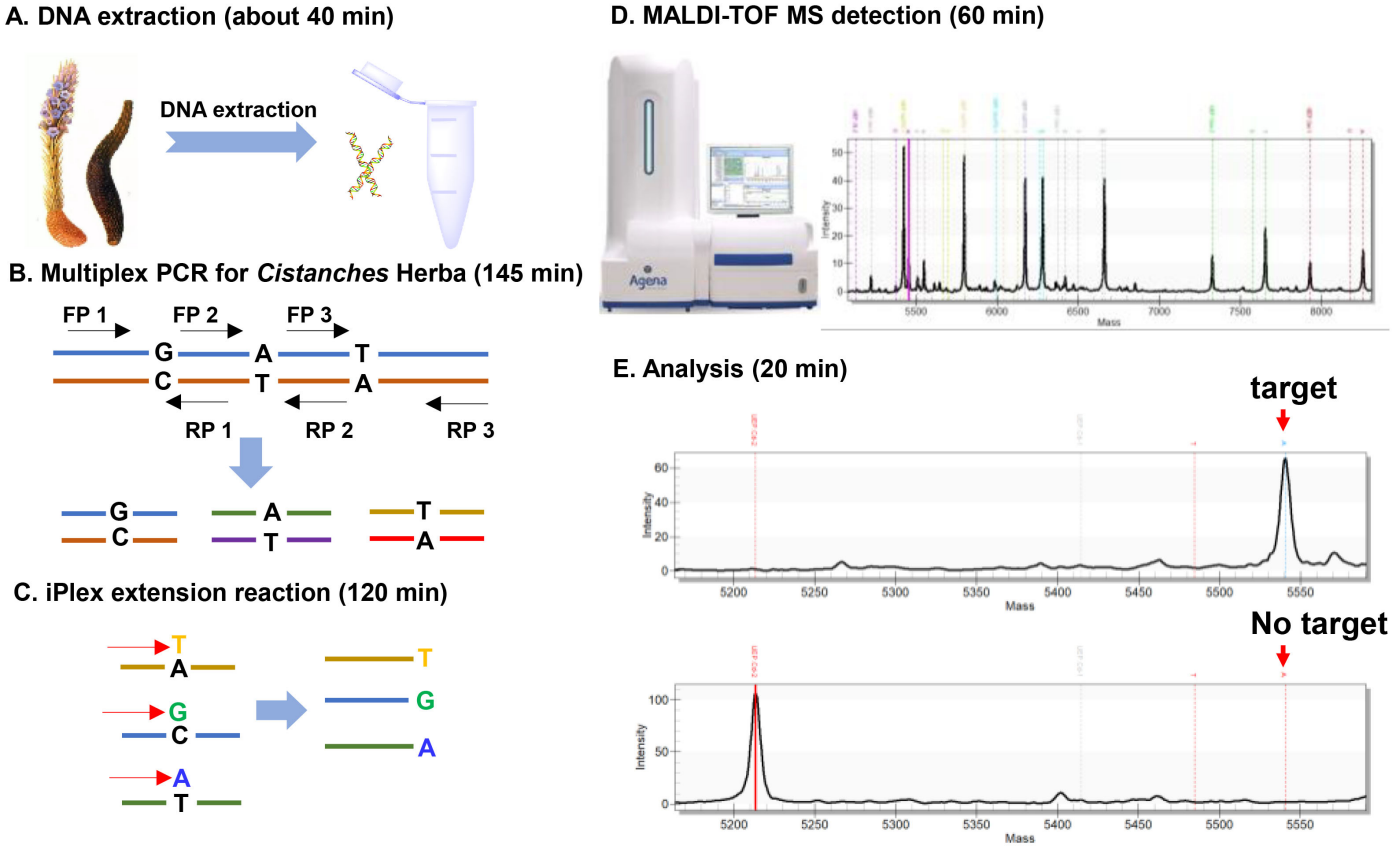
Schemic workflow of multiplex SNP-based authentication of *Cistanche* Herba using multiplex PCR and MALDI-TOF MS. (1) Sample preparation: fresh or dried suspect *Cistanche* species tissues are cryogenically ground, followed by genomic DNA extraction; (2) Multiplex PCR: amplification of target regions using species-discriminative primers; (3) Single-base extension: allele-specific extension probes hybridize and extend across SNP sites; (4) MS detection: purified extension products are analyzed by MALDI-TOF mass spectrometry; (5) Data analysis: TyperAnalyzer software performs automated peak clustering and SNP calling based on predefined SNP position.

To overcome the taxonomic challenge in authenticating rare pharmacopeial *C. deserticola* from *C. tubulosa* and other six adulterant species (*C. phelypaea, C. sinensis*, *C. ambigua*, *C. ridgewayana*, *C. salsa* and *C. rosea*), we developed a high-resolution MassARRAY SNP genotyping platform. The experimental workflow (**Figure 3**) consisted of the following key steps: (1) Collection of suspected *Cistanche* tissue samples and extraction of genomic DNA; (2) Multiplex PCR amplification of target regions of the extracted DNA; (3) Multiplex single-base extension (SBE) reaction using SAP-treated PCR products to generate mass-differentiated SBE products; (4) Detection of the SBE products via MALDI-TOF MS; and (5) Analysis of mass spectra using TyperAnalyzer 4.0 software to call SNPs for each target.

By integrating multiplex PCR with MALDI-TOF MS, this platform enables simultaneous detection and analysis of multiple SNPs across pharmacopeial *Cistanche* species. This integrated approach significantly improves authentication accuracy, while reducing detection time and reagent consumption. As a result, the proposed method shows strong potential for rapid and accurate identification of rare *Cistanche* materials, effectively preventing adulteration, ensuring therapeutic reliability, and supporting quality control in herbal product supply chains.

### Primers and probes development

To establish a molecular authentication system for *Cistanche* species, we developed a dual-locus SNP genotyping panel targeting nuclear ITS and chloroplast *rpl16* loci. The panel was constructed as follows: ITS sequences from six species (*C. deserticola* [KY753814.1], *C. tubulosa* [LT715486.1], *C. phelypaea* [KC480322.1], *C. sinensis* [LT715381.1], *C. ambigua* [LT715397.1], *C. ridgewayana* [LT715402.1], *C. rosea* [LT715457.1]) and *rpl16* sequences from four taxa (*C. deserticola* [AB116625.1], *C. tubulosa* [AB062415.1], *C. salsa* [AB062414.1], *C. sinensis* [AB116627.1]) were aligned using DNAMAN to identify species-specific diagnostic SNPs. These included two SNPs per species for *C. deserticola* (ITS: 482T, 585C; *rpl16*: 143A, 354A), *C. tubulosa* (*rpl16*: 98A, 270A), *C. salsa* (*rpl16*: 171A, 444C), and *C. sinensis* (*rpl16*: 107G, 320C), and one SNP for *C. phelypaea* (ITS: 560T), *C. ambigua* (ITS: 430C), *C. ridgewayana* (ITS: 61A), and *C. rosea* (ITS: 97T) (**Figures 1**, **2**). All the SNPs are unique to their respective species and are highlighted in green.

Based on these diagnostic SNPs, PCR primers were designed to span regions of high interspecific divergence, generating amplicons of 80-200 bp. A 10-mer tag (e.g., 5′-ACGTTGGATG-3′) was added to the 5′-end of each primer to increase its mass above 8,900 Da, thereby minimizing interference in a subsequent mass spectrometry analysis. Single-base extension probes were modified with heterologous nucleotide tags (6-12 nt) to produce mass-distinguishable extension products. However, due to the high genetic similarity among *Cistanche* species, the mass difference between some extension products were insufficient for reliable resolution by MALDI-TOF MS. To address this, the assay was divided into two multiplex pools: Tube 1 contained assays Ct-2, Csi-2, CpITS, CaITS, CroITS, CdITS-2, Csa-1, Csa-2, and Csi-1; Tube 2 contained Cd-2, Cd-1, CriITS, CdITS-1 and Ct-1 (**Tables 1**, **2**).

### Specificity validation of the MassARRAY genotyping platform for *Cistanche* Herba discrimination

**Figure 4:**
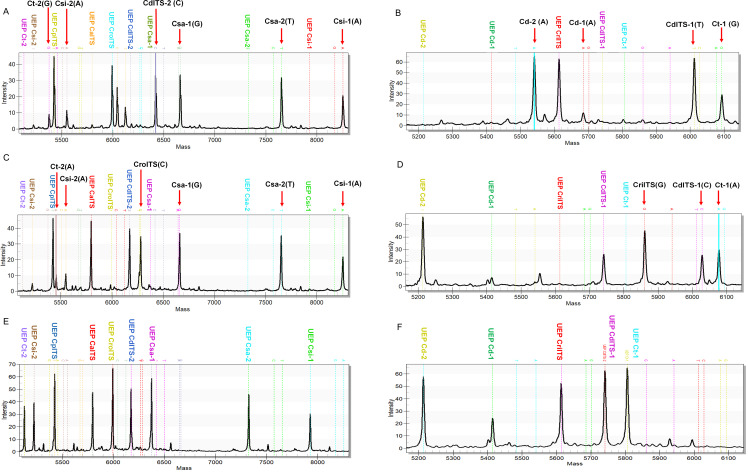
Representative MALDI-TOF mass spectra of single-base extension (SBE) products for the authentication of *C. deserticola* and *C. tubulosa*. **(A, C, E)** Results from Reaction 1 (Tube 1): **(A)***C. deserticola*, **(C)***C. tubulosa*, **(E)** nuclease-free water (negative control). **(B, D, F)** Results from Reaction 2 (Tube 2): **(B)***C. deserticola*, **(D)***C. tubulosa*, **(F)** nuclease-free water (negative control). Peaks correspond to the mass-to-charge ratios (m/z) of allele specific SBE products. Vertical dashed lines indicate the expected m/z positions for diagnostic SNPs. The absence of peaks in the negative controls confirms the specificity of the assay.

The specificity of the MassARRAY genotyping assay was evaluated using authenticated genomic DNA from *C. deserticola* and *C. tubulosa* through a dual-reaction MassARRAY profiling approach (**Figure 4**). For *C. deserticola*, Reaction 1 (**Figure 4A**) generated six distinct peaks. The CdITS-2 primer pair produced a ‘C’ allele (c.592C), confirming the identity of *C. deserticola.* The remaining five peaks were specific to *C. deserticola*, in contrast to the corresponding alleles in related *Cistanche* species: Ct-2 (G in *C. deserticola* vs. A in *C. tubulosa*), Csi-1(A in *C. deserticola* vs. G in *C. sinensis*), Csi-2 (A in *C. deserticola* vs. C in *C. sinensis*), Csa-1 (G in *C. deserticola* vs. A in *C. salsa*), and Csa-2 (T in *C. deserticola* vs. C in *C. salsa*). These results are consistent with the reference genomic profile of *C. deserticola* (NCBI Acession No.: KY753814.1, AB116625.1). In Reaction 2 (**Figure 4B**), four extension peaks were detected. Three of these, i.e. Cd-1 (c.354A), Cd-2 (c.143A); CdITS-1: c.482T) matched known *C. deserticola*-specific SNPs. The fourth peak from Ct-1, showed a ‘G’ allele (c.98G in *C. deserticola*) instead of the ‘A’ allele specific to *C. tubulosa* (c.98A), further confirming the assay’s resolution ability between these two species.

For *C. tubulosa* DNA, Reaction 1 (**Figure 4C**) yielded six peaks. The Ct-2 primer set (c.270A) exhibited 100% species fidelity, matching the *C. tubulosa* reference (NCBI No.: AB062415.1) (**Figure 2**). The other five peaks, i.e. Csi-1 (A in *C. tubulosa* vs. G in *C. sinensis*), Csi-2 (A in *C. tubulosa* vs. C in *C. sinensis*), CroITS (C in *C. tubulosa* vs. T in *C. rosea*), Csa-1 (G in *C. tubulosa* vs. A in *C. salsa*), Csa-2 (T in *C. tubulosa* vs. C in *C. salsa*) were also consistent with the *C. tubulosa* ITS (LT753809.1) (**Figure 1**) and rbl116 (AB062415.1) sequence (**Figure 2**). In Reaction 2 (**Figure 4D**), three peaks were observed: Ct-1 (c.97A) showed complete concordance with the *C. tubulosa*-specific *rpl16* sequence (NCBI No.: AB062415.1) (**Figure 2**), while CriITS (c.61G in *C. tubulosa* vs. A in *C. rosea*) and CdITS-1 (c.482C in *C. tubulosa* vs. T in *C. deserticola*) aligned with the *C. tubulosa* ITS profile (LT753809.1) (**Figure 1**). For the negative control without any *Cistanche* DNA sample, no SBE products were detected, but only unextended probe peaks were observed (**Figures 4E, F**).

These findings (**Table 3**) demonstrate that the platform achieves ability 100% specificity in discriminating *C. deserticola* and *C. tubulosa*, despite their high sequence homology (≈ 90%). For reliable authentication, *C. deserticola* is identified by the presence of CdITS-2 [C] in Reaction 1, and Cd-2 [A], Cd-1 [A], and CdITS-1 [T] in Reaction 2. *Cistanche tubulosa* is identified by Ct-2 [A] in Reaction 1 and Ct-1 [A] in Reaction 1.

**Table 3:** Detection results of *C. deserticola* and *C. tubulosa* using the MassARRAY.

Target gene	Designed SNP	Diagnostic SNP for *Cd*	Detected SNP in *Cd* gDNA	SNP in Cd sequence	Diagnostic SNP for *Ct*	Detected SNP in *Ct* DNA	SNP in *Ct* sequence
Cd-1	A	A	A	A		—	G
Cd-2	A	A	A	A		—	T
CdITS-1	T	T	T	T		C	C
CdITS-2	C	C	C	C		—	T
Ct-1	A		G	G	A	A	A
Ct-2	A		G	G	A	A	A
Csi-1	G		A	A		A	A
Csi-2	C		A	A		A	A
Csa-1	A		G	G		G	G
Csa-2	C		T	T		T	T
CpITS	T		—	T		—	T
CaITS_	C		—	−		—	T
CroITS_	T		—	C		C	C
CriITS	A		—	G		G	G

Green shading means nucleotide “A”; blue shading means nucleotide “T”; purple shading means nucleotide “G”; orange shading means nucleotide “C”.

### Sensitivity evaluation of the MassARRAY assay

The analytical sensitivity of the MassARRAY platform was evaluated using serial dilutions of *C. deserticola* genomic DNA mixed with a constant concentration of *C. tubulosa* genomic DNA. *Cistanche. deserticola* DNA was serially diluted 10-fold from 6.8 ng/μL to 6.8 pg/μL, and mixed at a 1:1 (v/v) ratio with *C. tubulosa* genomic DNA (9.4 ng/μL).

Target-specific SNPs showed 100% allelic concordance across all diluted levels of *C. deserticola* DNA (Cd-1: A; Cd-2: A; CdITS-1: T; CdITS-2: C) (**Table 4**). In mixed samples containing diluted *C. deserticola* DNA (0.68 ng/μL, 68 pg/μL and 6.8 pg/μL) and *C. tubulosa* DNA (9.4 ng/μL), both species were reliably detected using Reaction 1 and 2. The assay successfully identified *C. deserticola*-specific SNPs even at low concentration of 6.8 pg/μL DNA, corresponding to 0.07% *C. deserticola* DNA in the mixture, with no loss of allelic concordance. Concurrently, primers Ct-1 (A) and Ct-2 (A) consistently produced peaks specific to *C. tubulosa.*

**Table 4:** Analysis of *C. deserticola* and *C. tubulosa* in pure and mixed samples using the MassARRAY platform.

Target gene	Designed SNP	Pure Cd	0.68 ng Cd	68 pg Cd		6.8 pg Cd	Pure Ct	0.68ng Cd+Ct^a^		68 pg Cd+Ct^a^		6.8 pg Cd+Ct^a^
Cd-1	A	A	A	A		A	—	A		A		A
Cd-2	A	A	A	A		A	—	A		A		A
CdITS-1	T	T	T	T		T	C	T		T		T
CdITS-2	C	C	C	C		C	—	C		C		C
Ct-1	A	G	G	G		G	A	A	G	A	G	A
Ct-2	A	G	G	G		G	A	A	G	A		A
Csi-1	G	A	A	A		A	A	A		A		A
Csi-2	C	A	A	A		A	A	A		A		A
Csa-1	A	G	G	G		G	G	G		G		G
Csa-2	C	T	T	T		T	T	T		T		T
CpITS	T	—	—	—		—	—	—		—		—
CaITS_	C	—	—	C	T	T	—	C	T	T		T
CroITS_	T	—	—	—		—	C	C		C		C
CriITS	A	—	—	—		—	G	—		G		G

Ct^a^: the concentration of Ct is 9.4 ng/μL.

Green shading means nucleotide “A”; blue shading means nucleotide “T”; purple shading means nucleotide “G”; orange shading means nucleotide “C”.

Notably, in the mixture containing 0.68 ng/μL *C. deserticola* DNA and 9.4 ng/μL *C. tubulosa* DNA, both Ct-1 and Ct-2 assays produced double peaks (A/G), indicating the presence of both species. At a lower *C. deserticola* (68 pg/μL), only Ct-1 continued to yield double peaks (A/G), suggesting that Ct-1 exhibits higher sensitivity than Ct-2 for detecting *C. deserticola* in mixed samples.

All targeted *C. deserticola* genes, i.e. Cd-1, Cd-2, CdITS-1 and CdITS-2, correctly identified the corresponding SNPs across the entire dilution series of pure *C. deserticola* DNA (6.8 ng/μL-6.8 pg/μL) (**Supplementary Figures S1**-**S3**) and in all mixed samples with *C. tubulosa* (6.5% to 0.07% *C. deserticola* DNA content) (**Supplementary Figures S4**-**S6**). These results (**Table 4**) confirm the platform’s ability to detect *C. deserticola* DNA at concentrations as low as 0.07% in adulterated mixtures. Given that the DNA extraction protocol is consistent across *Cistanche* species, the assay is also expected to reliably identify trace amounts of *C. deserticola* in complex plant material.

### Field validation of the MassARRAY assay for actual samples

The MassARRAY assay was applied to genomic DNA extracted from homogenized tissue powder mixtures of *C. deserticola* and *C. tubulosa*. Results confirmed the detection of both *C. deserticola* and *C. tubulosa* in all the mixtures (**Supplementary Figures S7**-**S9**). Characteristic peaks for *C. deserticola—*Cd-2 [A], Cd-1 [A], and CdITS-1 [T] in Reaction 1, and CdITS-2 [C] in Reaction 2—were consistently observed across mixtures with varying proportions of *C. deserticola*. Similarly, diagnostic peaks for *C. tubulosa*—Ct-1 [A] in Reaction 1 and Ct-2 [A] in Reaction 2—were clearly present in all mixed samples (**Table 5**). Additional peaks corresponding to Csi-2 [A], CroITS [C], Csi-1 [G], Csa-2 [T], and Csi-1 [A] were also detected, consistent with reference SNP profiles of *C. deserticola* or *C. tubulosa* (**Figures 1**, **2**).

**Table 5:** Analysis of *C. deserticola* and *C. tubulosa* in artificial admixtures using the MassARRAY platform.

Target gene	Pure Cd	Pure Ct	20% Cd powder			10% Cd powder		1% Cd powder
Cd-1	A	—	A			A		A
Cd-2	A	—	A			A		A
CdITS-1	T	C	T			T		T
CdITS-2	C	—	C			C		C
Ct-1	G	A	A		G	A	G	A
Ct-2	G	A	A		G	A		A
Csi-1	A	A	A			A		A
Csi-2	A	A	A			A		A
Csa-1	G	G	G			G		G
Csa-2	T	T	T			T		T
CpITS	—	—	—			—		—
CaITS_	—	—	T	C		C		—
CroITS_	—	C	C			C		C
CriITS	—	G	G			G		G

Green shading means nucleotide “A”; blue shading means nucleotide “T”; purple shading means nucleotide “G”; orange shading means nucleotide “C”.

To further assess the field applicability of the assay, we analyzed 27 specimens morphically similar to *Cistanche* collected from five transcontinental regions (**Supplementary Table S1**, **Supplementary Figure S10**), and the results were summarized in **Table 6**. Genomic DNA from 22 specimens were successfully amplified with *Cistanche* genus-specific primers (Cis-F: CGGTAAATATGCTCTTCAAGC; Cis-R: GTCTATTCTTTCAATGCAAAGG) and subjected to Sanger sequencing and BLASTn analysis. However, high sequences similarity (> 97.58%) among closely related species led to ambiguous taxonomic assignments, with BLASTn returning multiple candidate species (**Supplementary Table S2**).

**Table 6:** Analysis of dried specimens using MassARRAY genotyping versus Sanger Sequencing.

No.	Cd-1		Cd-2	CdITS-1	CdITS-2		Ct-1		Ct-2		Csi-1	Csi-2	Csa-1	Csa-2	CpITS	CaITS	CroITS		CriITS	Sequencing results	MassArray
Design	A		A	T	C		A		A		G	C	A	C	T	C	T		A		
Cd	A		A	T	C		G		G		A	A	G	T	−	−	−		−	Cd	Cd
Ct	−		−	C	−		A		A		A	A	G	T	−	−	C		G	Ct	Ct
1	G		A	−	C	T	G		G		A	A	G	T	−	T	−		A	Csa, Cd	
2	−		−	C	−		A		A		A	A	G	T	−	−	C		G	Ct, Cp	Ct
3	−		−	C	−		A		A		A	A	G	T	−	−	C		G	Ct, Cp	Ct
4	−		−	C	−		A		A		A	A	G	T	−	−	T	C	G	Ct, Cp	Ct
5	G		A	C	C	T	G		G		A	A	G	T	−	T	−		A	Csa, Cd	
6	−		−	C	−		A		A		A	A	G	T	−	−	C		G	Ct, Cp	Ct
7	−		−	C	−		A		A		A	A	G	T	−	−	C		G	–	Ct
8	G		A	C	−		G		G		A	A	G	T	−	T	−		A	Csa, Cd	
9	−		A	C	−		A	G	A	G	A	−	G	−	−	−	C		−	Panax sp.	
10	G		A	C	C	T	G		G		A	A	G	T	−	T	−		A	Csa, Cd	
11	−		A	C	−		A		A		A	A	G	T	−	−	C		G	Ct, Cp	Ct
12	−		−	C	−		A		A		A	A	G	T	−	−	C		G	Ct, Cp	Ct
13	−		−	C	−		A		A		A	A	G	T	−	−	−		G	Ct, Cp	Ct
14	G		A	C	−		G		G		A	A	G	T	−	T	C		A	Csa, Cd	
15	G		−	C	−		A		A		A	A	G	T	−	−	C		−	Ct, Cp	Ct
16	G		A	C	C	T	G		G		A	A	G	T	−	T	−		A	Csa	
17	−		−	C	−		A		A		A	A	G	T	−	−	T	C	−	Csa, Cd	
18	−		−	C	−		A		A		A	A	G	T	−	−	−		G	–	
19	G		A	C	C	T	G		G		A	A	G	T	−	T	−		A	Ct, Cp	
20	G		A	C	C	T	G		G		A	A	G	T	−	T	−		A	Csa, Cd	
21	−		−	−	−		−		A		A	−	G	−	−	−	C		−	–	
22	−		−	−	C	T	−		G		A	A	G	T	−	T	−		−	Csa, Cd	
23	−		−	C	−		A		A		A	A	G	T	−	−	C		G	Ct, Cp	Ct
24	A	G	A	C	C	T	G		G		A	A	G	T	−	T	−		A	Ct, Cp	
25	G		A	C	C	T	G		G		A	A	G	T	−	T	−		A	Ct, Cp	
26	−		A	C	C		−		A		A	A	G	−	T	−	C		−	−	
27	−		A	C	−		A		A		A	A	G	−	T	−	C		G	−	Ct

Green shading means nucleotide “A”; blue shading means nucleotide “T”; purple shading means nucleotide “G”; orange shading means nucleotide “C”.

In comparison, MassARRAY analysis provided unambiguous species identification by matching detected SNPs to reference profiles of *C. deserticola* and *C. tubulosa*. Notably, 11 specimens (Sample 2-4, 6, 11-13, 15, 17, 23, 27) were conclusively identified as *C. tubulosa* by MassARRAY, whereas Sanger sequencing assigned these to *C. tubulosa* or *C. phelypae* (99.72% or 100% identity, E = 0) (**Supplementary Table S2**), underscoring the limitations of conventional sequencing in resolving closely related lineages.

Conversely, eight specimens (1, 5, 8, 10, 14, 17, 20, 22) sequenced as *C. salsa* (98.18% identity, E = 4e-160) or *C. deserticola* (97.58% identity, E = 9e-157) (**Supplementary Table S2**) showed incomplete SNP concordance with reference *C. deserticola* profiles in the MassARRAY assay. This result highlights the method’s enhanced capacity to detect specimens with potential introgression or hybridization events that are not readily distinguishable by sequence alignment alone.

## Discussion

### Challenges in quality control and authentication of *Cistanche* Herba

*Cistanche* Herba, a renowned herbal tonic with multifaceted pharmacologically active properties, serves as an integral component in traditional Chinese medicine (TCM). However, ensuring robust quality control in Chinese patent medicines remains challenging due to the inherent phytochemical complex of multi-component formulations and the intricate processing involved in production. Regulatory frameworks in many regions lack the rigor and harmonization needed to effectively monitor manufacturing practices, creating opportunities for economically motivated adulteration—such as substitution with inferior species (e.g. *Cistanche sinensis*), or intentional counterfeiting (Sun et al., 2012). Consequently, the development of reliable authentication methods is critical to ensuring drug safety and therapeutic efficacy (Moriya et al., 1995), particularly given the prevalence of adulteration in commercial markets.

Traditional quality control approaches, including microscopy, near-infrared reflectance spectroscopy (Zhang and Su, 2014; Zhang et al., 2015), liquid chromatography (Yao et al., 2015), and LC-MS profiling (Li et al., 2008; Wang et al., 2016; Zheng et al., 2014), are limited by overlapping phytochemical profiles among closely related species and their inability to detect low-abundance adulterants. While DNA barcoding and nucleotide signature analysis offer improved resolution, their reliance on sequencing and computational infrastructure limits accessibility in resource-limited settings (Wang et al., 2018).

### Comparison of molecular authentication technologies for TCM

When evaluating modern authentication techniques for TCM, the choice of platform must align with the specific application context. Each established method presents distinct advantages and limitations. The ability of analytical methods to differentiate closely related plant species depends on the types and content of secondary metabolite (Pant et al., 2021). Although high-performance liquid chromatography (HPLC)-based analytical methods are powerful for phytochemical standardization, they are often confounded by overlapping compound profiles among related species and cannot confirm genetic identity (Jiang et al., 2021; Li et al., 2019). In contrast, DNA is universal and unaffected by tissue type, processing, age, environmental factors or storage conditions (Gao et al., 2017; Wang et al., 2018), making DNA-based authentication increasing advocated by various Pharmacopoeia (Wu and Shaw, 2022).

DNA metabarcoding combines next-generation sequencing with barcoding to detect multiple taxa in complex mixtures (Coghlan et al., 2012). Although effective for identifying unknown adulterants, it requires substantial bioinformatic resources (Travadi et al., 2023; Zhou et al., 2025). Sequence-characterized Amplified Region (SCAR)-based PCR (SCAR-PCR) (Aradhana Yadav et al., 2012; Choi et al., 2008) and multiplex PCR (Travadi et al., 2022) are highly specific and technically accessible, but they are generally restricted to predefined targets and rely on low throughput agarose electrophoresis, limiting their effeteness against unknown or novel adulterants.

High-Resolution Melting (BAR-HRM) analysis detects subtle differences in PCR amplicon melting behavior influenced by GC content, length, and sequence. While effective for distinguishing distantly related species, HRM often lacks sufficient resolution and specificity to differentiate closely related *Cistanche* species such as *C. deserticola, C. tubulosa*, *C. sinensis*, which may differ by only a single nucleotide.

### Advantages of the MassARRAY platform for *Cistanche* authentication

The MassARRAY platform integrates multiplex PCR with MALDI-TOF MS, enabling single-nucleotide resolution and making it particularly suitable for authenticating *Cistanche* species (Almutawa et al., 2023; Wacharapluesadee et al., 2023). The platform allows simultaneous detection of up to 36 nucleic acid targets in a single run, enabling high-throughput screening of multiple SNPs without sequencing. This capability facilitates efficient and cost-effective detection of multiple potential adulterant species within a single sample.

Targeting the nuclear ITS and chloroplast *rpl16* loci, this dual-locus SNP panel leverages interspecific genetic divergence to achieve 100% specificity in distinguishing pharmacopeial *C. deserticola* and *C. tubulosa* from six adulterant species. Importantly, the authentication power of this method relies on a composite genotypic profile derived from multiple SNPs rather than any single diagnostic marker. Except of the diagnostic signals from CdITS-2 (C) with *C. deserticola* as the DNA template, signals from non-targeted probes, such as Csi-1 (A), Csi-2 (A), Csa-1 (G), Cas-2 (T), Ct-2 (G), reflect true genomic sequences rather than analytical errors, enabling confident exclusion of non-target species. The core strength of this approach lies precisely in its ability to generate and interpret these complex, composite profiles, which offer a higher level of specificity and reliability than methods based on individual markers. This multiplex profiling approach enhances specificity, reduces reagent consumption, and shortens processing time, addressing the urgent need for high-throughput screening in herbal supply chains.

### Detection of mixed genotypes and complex adulteration

A particularly significant advantage of this multi-SNP profiling approach is its ability to resolve complex genetic scenarios that confound conventional methods. In several commercial samples (e.g., Samples 1, 5, 8, 10, 14, 16, 17, 19, 20, 22), the MassARRAY system showed mixed signals, whereas Sanger sequencing produced ambiguous BLASTn results. Because Sanger sequencing generates an averaged consensus sequence from mixed DNA templates in a sample, it is ineffective for detecting hybridization or multi-species admixtures.

In contrast, MassARRAY simultaneously querying multiple discrete SNP loci, enabling detection and quantification of alleles from different species within a single sample. This capability provides direct evidence of hybridization events or complex adulterations, offering insights into population genetics and adulteration practices that are inaccessible through standard sequencing approaches.

### Selection of genetic markers for *Cistanche* species discrimination

Several plastid DNA regions, including chloroplast Maturase K (*mat*K), ribulose-bisphosphate carboxylase (*rbc*L), chloroplast *psb*A-*trn*H region and the nuclear internal transcribed spacer (ITS), have been recommended as the barcode for plant discrimination (Fazekas et al., 2008; Zhang and Jiang, 2020). While *mat*K and *psb*A-*trn*H evolve rapidly, ITS is relatively conserved (Hollingsworth et al., 2011). The *rbcL* gene, encoding the large subunit of ribulose-bisphosphate carboxylase, has been used for phylogenetic analysis among plant species (Bell et al., 2017) but has been reported to be lost in some plants. In contrast, the plastid *rpl16* gene is retained even in non-photosynthetic holoparasitic plant (Wolfe et al., 1992) and has been successfully used to resolve phylogenetic relationship among *C. deserticola, C. salsa and C. tubulosa* (Tomari et al., 2002). This biological stability makes *rpl16* particularly suitable for authenticating *Cistanche* Herba.

### Importance of short amplicons for processed herbal products

Industrial processing, including high-temperature drying, mechanical grinding, solvent extraction, and sterilization, severely fragments genomic DNA in herbal products. Although ITS/ITS2 regions are widely accepted molecular markers for botanical authentication, their application is considerably limited in highly processed materials due to DNA degradation (de Boer et al., 2015; Newmaster et al., 2013). Conventional PCR assays targeting long amplicons (> 400 bp) often fail under such conditions, since the probability of amplifying an intact DNA template spanning the entire region is significantly reduce (Wang et al., 2016). This limitation hinders the utility of these methods for quality control of processed herbal formulations such as powders and decoctions. By contrast, assays targeting short amplicons (typically under 200 bp) significantly improve amplification success in degraded samples. The MassARRAY platform adopts this principle by generating amplicons of 80-200 bp, enabling reliable authentication of processed herbal materials.

### Specificity, sensitivity and market implications

The specificity of the MassARRAY assay was validated using authenticated genomic DNA from *C. deserticola* and *C. tubulosa*. Adulteration of *Cistanche* Herba is driven by market dynamics. *Cistanche deserticola*, the sole species traditionally recognized as authentic *Cistanche* Herba in the Chinese Phamacopoeia Commission (2000) (Chinese Pharmacopoeia, 2000), has historically faced supply shortages, leading to the inclusion of *C. tubulosa* as a supplementary species since 2005 (Jiang and Tu, 2009). Given the significant prices disparity between these species with *C. deserticola* commanding a premium, we tested the assay’s sensitivity using *C. deserticola*/*C. tubulosa* mixtures. Notably, primers such as Ct-1 detected a ‘G’ allele in *C. deserticola* instead of the expected ‘A’ allele designed for *C. tubulosa*, aligning with the *C. deserticola* reference sequence (NCBI: AB116625.1]). This underscores the assay’s capacity to resolve SNPs in conserved regions despite high sequence similarity. Sensitivity testing revealed a detection limit of 0.07% *C. deserticola* DNA in *C. tubulosa* mixtures, demonstrating the assay’s potential to identify trace adulterates. The dynamic range (6.8 ng/μL to 6.8 pg/μL) and robustness in diluted samples further highlight its applicability to degraded materials, a common challenge in processed herbal products. These findings align with prior studies demonstrating MassARRAY’s efficacy in detecting low-abundance targets in complex matrices, including cancer mutations and viral subtyping (Almutawa et al., 2023; Min et al., 2016).

### Broader implications and future directions

Adulteration risks extend beyond species substitution. *Cistanche sinensis*, a common adulterant, contains similar bioactive compounds (e.g. echinacoside and acteoside) with authentic *Cistanche* Herba but is significantly less expensive (Liu et al., 2013), creating. strong incentives for illicit use. The MassARRAY platform directly addresses these concerns by enabling rapid, precise authentication of pharmacopeial species, supporting compliance with Convention on International Trade in Endangered Species (CITES) regulations and pharmacopeial standards. Its ability to detect trace adulterants at 0.07% concentrations could deter intentional dilution practices, enhancing consumer safety and market integrity.

Future work should expanding the SNP panel to include mitochondrial genes or hypervariable chloroplast regions to improve resolution for underrepresent adulterants. Additionally, systematic evaluation of DNA degradation thresholds in highly processed samples (e.g. extracts, decoctions) and optimization of pre-amplification strategies will be essential for establishing a scalable platform applicable to functional foods and traditional Chinese medicine products. These advancements will further solidify MassARRAY technology as a cornerstone for global quality assurance of health food supplement and medicinal herb globally.

## Conclusion

This study successfully developed and validated an innovative MassARRAY-based SNP genotyping platform for authenticating *Cistanche* Herba, addressing a critical need in quality control of traditional medicines. The method demonstrates exceptional technical performance, achieving 100% specificity in distinguishing *Cistanche* species from adulterants and a remarkable sensitivity to detect trace adulteration as low as 0.07% *C. deserticola* genomic DNA. A key innovation lies in its reliance on a multi-SNP composite profile, which not only provide a more reliable authentication outcome than single-marker methods but also uniquely enables the detection of complex genetic events like hybridization, a common challenge that confounds conventional Sanger sequencing. By integrating high specificity, sensitivity and throughput into a single, cost-effective workflow capable of analyzing endangered *Cistanche* herba. This platform offers a scalable solution for combating herbal adulteration in functional foods. Future expansion of the SNP database and further validation will further solidify its role in ensuring global supply chain integrity, protecting consumer safety, and supporting the conservation of valuable medicinal species.

## Data Availability

The original contributions presented in the study are included in the article/**Supplementary Material**. Further inquiries can be directed to the corresponding authors.
