# Supplementary material for: Rapid authentication of endangered Cistanche Herba (Rou Cong Rong) using a high-throughput multi-SNP panel and MALDI-TOF MS platform
Source: Front Plant Sci. 2026 Jan 26;16:1677826. doi: 10.3389/fpls.2025.1677826 (PMC12884972; doi:10.3389/fpls.2025.1677826)
Supplement: Supplementary file 1 [file DataSheet1.doc]

**Rapid Authentication of Endangered *Cistanche* Herba (*Rou Cong Rong*) Based on Multi-SNP/MALDI-TOF MS Platform**

**Rong Lei^a^, Yixia Cao^c^, Yifen Yang^b^, Haolong Cong^a^, Limei Li^d^, Xin Li^e*^, Junxia Shi^b*^**

*^a^ Chinese Academy of Quality and Inspection & Testing, Beijing, 100176, China*

*^b^ Technology Center of Chengdu Customs District, Chengdu, Sichuan 610041, China*

*^c^* *China Certification & Inspection Group, Beijing 100028, China*

*^d^ Jilin Provincial Academy of Forestry Science, Changchun, 130033, China*

*^e^ Technology Center of Dalian Customs District, Dalian, 116001, China*

Table S1 Information of samples submitted to Airport Customs

| No. | **Code** | **Inspection number** | **Results by Customs** | Customs Name | Source |
| --- | --- | --- | --- | --- | --- |
| 1 | 2537 | 2311790001002537 | Cistanche sp. | Shuangliu Airport Customs | Uzbekistan |
| 2 | 2473 | 2311790001002473 | Cistanche sp. | Shuangliu Airport Customs | Egypt |
| 3 | 0708 | 2311790001000708 | Cistanche sp. | Shuangliu Airport Customs | The United Arab Emirates |
| 4 | 2649 | 2311790001002649 | Cistanche sp. | Shuangliu Airport Customs | The United Arab Emirates |
| 5 | 0017 | 2411790001000017 | Cistanche sp. | Shuangliu Airport Customs | Uzbekistan |
| 6 | 0645 | 2311790001000645 | Cistanche sp. | Shuangliu Airport Customs | Ethiopia |
| 7 | 0644 | 2311790001000644 | Cistanche sp. | Shuangliu Airport Customs | The United Arab Emirates |
| 8 | 2536 | 2311790001002536 | Cistanche sp. | Shuangliu Airport Customs | Uzbekistan |
| 9 | 2691-1 | 2311790001002691-1 | Cistanche sp. | Shuangliu Airport Customs | Ethiopia |
| 10 | 2540 | 2311790001002540 | Cistanche sp. | Shuangliu Airport Customs | Uzbekistan |
| 11 | 0899 | 2311790001000899 | Cistanche sp. | Shuangliu Airport Customs | The United Arab Emirates |
| 12 | 0418 | 2311790001000418 | Cistanche sp. | Shuangliu Airport Customs | The United Arab Emirates |
| 13 | 0668 | 2311790001000668 | Cistanche tubulosa | Shuangliu Airport Customs | The United Arab Emirates |
| 14 | 2690 | 2311790001002690 | Cistanche sp. | Shuangliu Airport Customs | Uzbekistan |
| 15 | 2691-2 | 2311790001002691-2 | Cistanche sp. | Shuangliu Airport Customs | Ethiopia |
| 16 | 2539 | 2311790001002539 | Cistanche sp. | Shuangliu Airport Customs | Uzbekistan |
| 17 | 0535 | 2311790001000535 | Cistanche sp. | Shuangliu Airport Customs | The United Arab Emirates |
| 18 | 2475 | 2311790001002475 | Cistanche sp. | Shuangliu Airport Customs | The United Arab Emirates |
| 19 | 0018 | 2411790001000018 | Cistanche sp. | Shuangliu Airport Customs | Uzbekistan |
| 20 | 2538 | 2311790001002538 | Cistanche sp. | Shuangliu Airport Customs | Uzbekistan |
| 21 | 0898 | 2311790001000898 | Cistanche sp. | Shuangliu Airport Customs | The United Arab Emirates |
| 22 | 2039 | 2311790001002039 | Cistanche sp. | Shuangliu Airport Customs | Uzbekistan |
| 23 | 0804 | 2311790001000804 | Cistanche tubulosa | Shuangliu Airport Customs | Shuangliu Airport Customs |
| 24 | 2896 | 2311790001002896 | Cistanche sp. | Shuangliu Airport Customs | Uzbekistan |
| 25 | 2474 | 23117900010002474 | Cistanche sp. | Shuangliu Airport Customs | Uzbekistan |
| 26 | 0536 | 2311790001000536 | Cynomorium coccineum | Shuangliu Airport Customs | The United Arab Emirates |
| 27 | 0709 | 2311790001000709 | Cistanche sp. | Shuangliu Airport Customs | The United Arab Emirates |

Table S2. Sequencing results of samples in Table S1.

| **No.** | **Sample Code** | **Sequence (5'-3')** | **Sanger sequencing results** | **MassARRAY** |
| --- | --- | --- | --- | --- |
| 1 | 2537 | GGAAAAATATGGGTCCGTATATTTCCAGATAAACCAGTTACAGTAAGACCAGCCGAAACACGTATGGGTTCGGGGAAAGGAGTCCCCGAATATTGGGTAGCCGTTGTTAAACCGGAACAAATACTATATGAAATGGGCGGAGTAACCGAAAATATAGCTAGACGAGCTATTTTAATAGCAGCATCAAAAATGCCTATACGAACTCAATTTATTATTTCTGGATAAATAATGTAGAAACGACAAAAATGAGTCTTGGTATGAAACAAAAGCCCAGGCTTCTTTTTTAGACAAACAATATTTCTTTTTATTTTATTCATCCTTTGCATTGAA | *C. salsa,* 98.18% (E value: 4e-160);  *C. deserticola,* 97.58% (E value: 9e-157); |  |
| 2 | 2473 | GGCGAGCAATGACACGAAATGCACGCCGTGGTGGAAAAATTTTTGTCCGTATATTTCCAGATAAACCAGTTACAGTAAGACCAGCTGAAACACGTATGGGTTCGGGGAAAGGATCCCCCGAATATTGGGTAGCTGTTGTTAAACCGAAACGAATACTATATGAAATGAGCGGAGTAACCGAAAATATAGCTAGACGAGCTATTTTAATAGCAGCATCAAAAATGCCTATACGAACTCAATTTATTATTTCTGGATAAAAATGTAGAAACAACAAAAATGAGTCTTGGTATGAAACAAAAACCCAGGTTTCTTTTTTTAGACAAACAATATTTCTTTTATTTTATTCATCCTTTGCATTG | *C. tubulosa,* 100% (E value 0)  *C. phelypaea,* 100% (E value 0) | *C. tubulosa,* |
| 3 | 0708 | CGGCGAGCAATGACACGAAATGCACGCCGTGGTGGAAAAATTTTTGTCCGTATATTTCCAAATAAACCAGTTACAGTAAGACCAGCTGAAACACGTATGGGTTCGGGGAAAGGATCCCCCGAATATTGGGTAGCTGTTGTTAAACCGAAACGAATACTATATGAAATGAGCGGAGTAACCGAAAATATAGCTAGACGAGCTATTTTAATAGCAGCATCAAAAATGCCTATACGAACTCAATTTATTATTTCTGGATAAAAATGTAGAAACAACAAAAATGAGTCTTGGTATGAAACAAAAACCCAGGTTTCTTTTTTTAGACAAACAATATTTCTTTTATTTTATTCATCCTTTGCATTGAA | *C. tubulosa,* 99.72 (E value 0)  *C. phelypaea,* 99.72 (E value 0) | *C. tubulosa,* |
| 4 | 2649 | CGGCGAGCAATGACACGAAATGCACGCCGTGGTGGAAAAATTTTTGTCCGTATATTTCCAGATAAACCAGTTACAGTAAGACCAGCTGAAACACGTATGGGTTCGGGGAAAGGATCCCCCGAATATTGGGTAGCTGTTGTTAAACCGAAACGAATACTATATGAAATGAGCGGAGTAACCGAAAATATAGCTAGACGAGCTATTTTAATAGCAGCATCAAAAATGCCTATACGAACTCAATTTATTATTTCTGGATAAAAATGTAGAAACAACAAAAATGAGTCTTGGTATGAAACAAAAACCCAGGTTTCTTTTTTTAGACAAACAATATTTCTTTTATTTTATTCATCCTTTGCATTG | *C. tubulosa,* 100% (E value 0)  *C. phelypaea,* 100% (E value 0) | *C. tubulosa,* |
| 5 | 0017 | GGAAAAATATGGGTCCGTATATTTCCAGATAAACCAGTTACAGTAAGACCAGCCGAAACACGTATGGGTTCGGGGAAAGGAGTCCCCGAATATTGGGTAGCCGTTGTTAAACCGGAACAAATACTATATGAAATGGGCGGAGTAACCGAAAATATAGCTAGACGAGCTATTTTAATAGCAGCATCAAAAATGCCTATACGAACTCAATTTATTATTTCTGGATAAATAATGTAGAAACGACAAAAATGAGTCTTGGTATGAAACAAAAGCCCAGGCTTCTTTTTTAGACAAACAATATTTCTTTTTATTTTATTCATCCTTTGCATTGAA | *C. salsa,* 98.18% (E value: 4e-160);  *C. deserticola,* 97.58% (E value: 9e-157); |  |
| 6 | 0645 | GGCGAGCAATGACACGAAATGCACGCCGTGGTGGAAAAATTTTTGTCCGTATATTTCCAGATAAACCAGTTACAGTAAGACCAGCTGAAACACGTATGGGTTCGGGGAAAGGATCCCCCGAATATTGGGTAGCTGTTGTTAAACCGAAACGAATACTATATGAAATGAGCGGAGTAACCGAAAATATAGCTAGACGAGCTATTTTAATAGCAGCATCAAAAATGCCTATACGAACTCAATTTATTATTTCTGGATAAAAATGTAGAAACAACAAAAATGAGTCTTGGTATGAAACAAAAACCCAGGTTTCTTTTTTTAGACAAACAATATTTCTTTTATTTTATTCATCCTTTGCATTGA | *C. tubulosa,* 100% (E value 0)  *C. phelypaea*, 100% (E value 0) | *C. tubulosa,* |
| 8 | 2536 | TGGAAAAATATGGGTCCGTATATTTCCAGATAAACCAGTTACAGTAAGACCAGCCGAAACACGTATGGGTTCGGGGAAAGGAGTCCCCGAATATTGGGTAGCCGTTGTTAAACCGGAACAAATACTATATGAAATGGGCGGAGTAACCGAAAATATAGCTAGACGAGCTATTTTAATAGCAGCATCAAAAATGCCTATACGAACTCAATTTATTATTTCTGGATAAATAATGTAGAAACGACAAAAATGAGTCTTGGTATGAAACAAAAGCCCAGGCTTCTTTTTTAGACAAACAATATTTCTTTTTATTTTATTCATCCTTTGCATTGAA | *C. salsa,* 98.19% (E value: 1e-160)*;*  *C. deserticola,* 97.59% (E value: 3e-157); |  |
| 9 | 2691-1 | CGACGAGCAATGACACGAAATGCACGACGTGGTGGAAAAATATGGGTACGTATATTTCCAGACAAACCGGTTACAGTAAGACCCGCAGAAACACGTATGGGTTCGGGGAAAGGATCCCCTGAATATTGGGTAGCTGTTGTTAAACCAGGTCGAATCCTTTATGAAATGGGTGGAGTAACAGAAAATATAGCCAGAAAGGCTATTTCAATAGCATCGTCTAAAATGCCTATACGAACTCAATTCATTATTTCGGCATAAAAATGGAGAATCAAAGGAAATAGGTCTTGAGAATTAAAAAAAAAA | *Penax. 100.00%* (E value: 4e-155); |  |
| 10 | 2540 | GGAAAAATATGGGTCCGTATATTTCCAGATAAACCAGTTACAGTAAGACCAGCCGAAACACGTATGGGTTCGGGGAAAGGAGTCCCCGAATATTGGGTAGCCGTTGTTAAACCGGAACAAATACTATATGAAATGGGCGGAGTAACCGAAAATATAGCTAGACGAGCTATTTTAATAGCAGCATCAAAAATGCCTATACGAACTCAATTTATTATTTCTGGATAAATAATGTAGAAACGACAAAAATGAGTCTTGGTATGAAACAAAAGCCCAGGCTTCTTTTTTAGACAAACAATATTTCTTTTTATTTTATTCATCCTTTGCATTGAA | *C. salsa, 98.18%* (E value: 4e-160);  *C. deserticola,* 97.58% (E value: 9e-157); |  |
| 11 | 0899 | CGGCGAGCAATGACACGAAATGCACGCCGTGGTGGAAAAATTTTTGTCCGTATATTTCCAGATAAACCAGTTACAGTAAGACCAGCTGAAACACGTATGGGTTCGGGGAAAGGATCCCCCGAATATTGGGTAGCTGTTGTTAAACCGAAACGAATACTATATGAAATGAGCGGAGTAACCGAAAATATAGCTAGACGAGCTATTTTAATAGCAGCATCAAAAATGCCTATACGAACTCAATTTATTATTTCTGGATAAAAATGTAGAAATAACAAAAATGAGTCTTGGTATGAAACAAAAACCCAGGTTTCTTTTTTTAGACAAACAATATTTCTTTTATTTTATTCATCCTTTGCATTGA | *C. tubulosa,*99.72 % (E value 0)  *C. phelypaea,* 99.72 % (E value 0) | *C. tubulosa,* |
| 12 | 0418 | CGGCGAGCAATGACACGAAATGCACGCCGTGGTGGAAAAATTTTTGTCCGTATATTTCCAGATAAACCAGTTACAGTAAGACCAGCTGAAACACGTATGGGTTCGGGGAAAGGATCCCCCGAATATTGGGTAGCTGTTGTTAAACCGAAACGAATACTATATGAAATGAGCGGAGTAACCGAAAATATAGCTAGACGAGCTATTTTAATAGCAGCATCAAAAATGCCTATACGAACTCAATTTATTATTTCTGGATAAAAATGTAGAAACAACAAAAATGAGTCTTGGTATGAAACAAAAACCCAGGTTTCTTTTTTTAGACAAACAATATTTCTTTTATTTTATTCATCCTTTGCATTGA | *C. tubulosa,* 100% (E value 0)  *C. phelypaea,* 100% (E value 0) | *C. tubulosa,* |
| 13 | 0668 | CGGCGAGCAATGACACGAAATGCACGCCGTGGTGGAAAAATTTTTGTCCGTATATTTCCAGATAAACCAGTTACAGTAAGACCAGCTGAAACACGTATGGGTTCGGGGAAAGGATCCCCCGAATATTGGGTAGCTGTTGTTAAACCGAAACGAATACTATATGAAATGAGCGGAGTAACCGAAAATATAGCTAGACGAGCTATTTTAATAGCAGCATCAAAAATGCCTATACGAACTCAATTTATTATTTCTGGATAAAAATGTAGAAACAACAAAAATGAGTCTTGGTATGAAACAAAAACCCAGGTTTCTTTTTTTAGACAAACAATATTTCTTTTATTTTATTCATCCTTTGCATTG | *C. tubulosa,* 100% (E value 0)  *C. phelypaea,* 100% (E value 0) | *C. tubulosa,* |
| 14 | 2690 | TGGAAAAATATGGGTCCGTATATTTCCAGATAAACCAGTTACAGTAAGACCAGCCGAAACACGTATGGGTTCGGGGAAAGGAGTCCCCGAATATTGGGTAGCCGTTGTTAAACCGGAACAAATACTATATGAAATGGGCGGAGTAACCGAAAATATAGCTAGACGAGCTATTTTAATAGCAGCATCAAAAATGCCTATACGAACTCAATTTATTATTTCTGGATAAATAATGTAGAAACGACAAAAATGAGTCTTGGTATGAAACAAAAGCCCAGGCTTCTTTTTTAGACAAACAATATTTCTTTTTATTTTATTCATCCTTTGCATTGAA | *C. salsa,* 98.19% (E value: 1e-160);  *C. deserticola,* 97.59% (E value: 3e-157); |  |
| 15 | 2691-2 | CGGCGAGCAATGACACGAAATGCACGCCGTGGTGGAAAAATTTTTGTCCGTATATTTCCAGATAAACCAGTTACAGTAAGACCAGCTGAAACACGTATGGGTTCGGGGAAAGGATCCCCCGAATATTGGGTAGCTGTTGTTAAACCGAAACGAATACTATATGAAATGAGCGGAGTAACCGAAAATATAGCTAGACGAGCTATTTTAATAGCAGCATCAAAAATGCCTATACGAACTCAATTTATTATTTCTGGATAAAAATGTAGAAACAACAAAAATGAGTCTTGGTATGAAACAAAAACCCAGGTTTCTTTTTTTAGACAAACAATATTTCTTTTATTTTATTCATCCTTTGCATTGAA | *C. tubulosa,* 100% (E value 0)；  *C. phelypaea,* 100% (E value 0) | *C. tubulosa,* |
| 16 | 2539 | GAAAAATATGGGTCCGTATATTTCCAGATAAACCAGTTACAGTAAGACCAGCCGAAACACGTATGGGTTCGGGGAAAGGAGTCCCCGAATATTGGGTAGCCGTTGTTAAACCGGAACAAATACTATATGAAATGGGCGGAGTAACCGAAAATATAGCTAGACGAGCTATTTTAATAGCAGCATCAAAAATGCCTATACGAACTCAATTTATTATTTCTGGATAAATAATGTAGAAACGACAAAAATGAGTCTTGGTATGAAACAAAAGCCCAGGCTTCTTTTTTAGACAAACAATATTTCTTTTTATTTTATTCATCCTTTGCATTGAA | *C. salsa,* 98.18% (E value: 1e-159);  *C. deserticola,* 97.59% (E value: 3e-156); |  |
| 17 | 0535 | CGGCGAGCAATGACACGAAATGCACGCCGTGGTGGAAAAATTTTTGTCCGTATATTTCCAGATAAACCAGTTACAGTAAGACCAGCTGAAACACGTATGGGTTCGGGGAAAGGATCCCCCGAATATTGGGTAGCTGTTGTTAAACCGAAACGAATACTATATGAAATGAGCGGAGTAACCGAAAATATAGCTAGACGAGCTATTTTAATAGCAGCATCAAAAATGCCTATACGAACTCAATTTATTATTTCTGGATAAAAATGTAGAAACAACAAAAATGAGTCTTGGTATGAAACAAAAACCCAGGTTTCTTTTTTTAGACAAACAATATTTCTTTTATTTTATTCATCCTTTGCATTGAA | *C. tubulosa,* 100% (E value 0)  *C. phelypaea,* 100% (E value 0) | *C. tubulosa,* |
| 19 | 0018 | GGCGAGCAATGACACGAAATGCACGCCGTGGTGGAAAAATTTTTGTCCGTATATTTCCAGATAAACCAGTTACAGTAAGACCAGCTGAAACACGTATGGGTTCGGGGAAAGGATCCCCCGAATATTGGGTAGCTGTTGTTAAACCGAAACGAATACTATATGAAATGAGTGGAGTAACCGAAAATATAGCTAGACGAGCTATTTTAATAGCAGCATCAAAAATGCCTATACGAACTCAATTTATTATTTCTGGATAAAAATGTAGAAACAACAAAAATGAGTCTTGGTATGAAACAAAAACCCAGGTTTCTTTTTTTAGACAAACAATATTTCTTTTATTTTATTCATCCTTTGCATTGAA | *C. tubulosa,* 99.72% (E value 0)  *C. phelypaea,* 99.72% (E value 0) |  |
| 20 | 2538 | GCGAGTCGACGAGCAATGACACGAAATGCACGCCGTGGTGGAAAAATATGGGTCCGTATATTTCCAGATAAACCAGTTACAGTAAGACCAGCCGAAACACGTATGGGTTCGGGGAAAGGAGTCCCCGAATATTGGGTAGCCGTTGTTAAACCGGAACAAATACTATATGAAATGGGCGGAGTAACCGAAAATATAGCTAGACGAGCTATTTTAATAGCAGCATCAAAAATGCCTATACGAACTCAATTTATTATTTCTGGATAAATAATGTAGAAACGACAAAAATGAGTCTTGGTATGAAACAAAAGCCCAGGCTTCTTTTTTAGACAAACAATATTTCTTTTTATTTTATTCATCCTTTGCATTGAA | *C. salsa,* 98.10% (E value: 5e-180);  *C. deserticola,* 97.57% (E value: 1e-176); |  |
| 22 | 2039 | GCGAGTCGACGAGCAATGACACGAAATGCACGCCGTGGTGGAAAAATATGGGTCCGTATATTTCCAGATAAACCAGTTACAGTAAGACCAGCCGAAACACGTATGGGTTCGGGGAAAGGAGTCCCCGAATATTGGGTAGCCGTTGTTAAACCGGAACAAATACTATATGAAATGGGCGGAGTAACCGAAAATATAGCTAGACGAGCTATTTTAATAGCAGCATCAAAAATGCCTATACGAACTCAATTTATTATTTCTGGATAAATAATGTAGAAACGACAAAAATGAGTCTTGGTATGAAACAAAAGCCCAGGCTTCTTTTTTAGACAAACAATATTTCTTTTTATTTTATTCATCCTTTGCATTGAA | *C. salsa,* 98.10% (E value: 5e-180);  *C. deserticola,* 97.57% (E value: 1e-176); |  |
| 23 | 0804 | CGGCGAGCAATGACACGAAATGCACGCCGTGGTGGAAAAATTTTTGTCCGTATATTTCCAGATAAACCAGTTACAGTAAGACCAGCTGAAACACGTATGGGTTCGGGGAAAGGATCCCCCGAATATTGGGTAGCTGTTGTTAAACCGAAACGAATACTATATGAAATGAGCGGAGTAACCGAAAATATAGCTAGACGAGCTATTTTAATAGCAGCATCAAAAATGCCTATACGAACTCAATTTATTATTTCTGAATAAAAATGTAGAAACAACAAAAATGAGTCTTGGTATGAAACAAAAACCCAGGTTTCTTTTTTTAGACAAACAATATTTCTTTTATTTTATTCATCCTTTGCATTGAA | *C. tubulosa,* 99.72% (E value 0)  *C. phelypaea,* 99.72% (E value 0) | *C. tubulosa,* |
| 24 | 2896 | GCGAGTCGACGAGCAATGACACGAAATGCACGCCGTGGTGGAAAAATATGGGTCCGTATATTTCCAGATAAACCAGTTACAGTAAGACCAGCCGAAACACGTATGGGTTCGGGGAAAGGAGTCCCCGAATATTGGGTAGCCGTTGTTAAACCGGAACAAATACTATATGAAATGGGCGGAGTAACCGAAAATATAGCTAGACGAGCTATTTTAATAGCAGCATCAAAAATGCCTATACGAACTCAATTTATTATTTCTGGATAAATAATGTAGAAACGACAAAAATGAGTCTTGGTATGAAACAAAAGCCCAGGCTTCTTTTTTAGACAAACAATATTTCTTTTTATTTTATTCATCCTTTGCATTGAAA | *C. salsa,* 98.11% (E value: 1e-180);  *C. deserticola,* 97.57% (E value: 3e-177); |  |
| 25 | 2474 | GCGAGTCGACGAGCAATGACACGAAATGCACGCCGTGGTGGAAAAATATGGGTCCGTATATTTCCAGATAAACCAGTTACAGTAAGACCAGCCGAAACACGTATGGGTTCGGGGAAAGGAGTCCCCGAATATTGGGTAGCCGTTGTTAAACCGGAACAAATACTATATGAAATGGGCGGAGTAACCGAAAATATAGCTAGACGAGCTATTTTAATAGCAGCATCAAAAATGCCTATACGAACTCAATTTATTATTTCTGGATAAATAATGTAGAAACGACAAAAATGAGTCTTGGTATGAAACAAAAGCCCAGGCTTCTTTTTTAGACAAACAATATTTCTTTTTATTTTATTCATCCTTTGCATTGA | *C. salsa,* 98.101% (E value: 2e-179);  *C. deserticola,* 97.56% (E value: 4e-176); |  |

**FIGURE S1 **. MALDI-TOF mass spectra of single-base extension (SBE) products from 0.68 ng/μL *C. deserticola* DNA using reaction 1 and reaction 2.

**FIGURE S2 **. MALDI-TOF mass spectra of single-base extension (SBE) products from 68 pg/μL *C. deserticola* DNA using reaction 1 and reaction 2.

**FIGURE S3 ** MALDI-TOF mass spectra of single-base extension (SBE) products from 6.8 pg/μL *C. deserticola* DNA using reaction 1 and reaction 2.

**FIGURE S4 **. MALDI-TOF mass spectra of single-base extension (SBE) products from 0.68 ng/μL *C. deserticola* DNA and 9.4 ng/μL *C. tubulosa* DNA using reaction 1 and reaction 2.

**FIGURE S5 ** MALDI-TOF mass spectra of single-base extension (SBE) products from 68 pg/μL *C. deserticola* DNA and 9.4 ng/μL *C. tubulosa* DNA using reaction 1 and reaction 2.

**FIGURE S6**. MALDI-TOF mass spectra of single-base extension (SBE) products from 6.8 pg/μL *C. deserticola* DNA and 9.4 ng/μL *C. tubulosa* DNA using reaction 1 and reaction 2.

**FIGURE S7 ** MALDI-TOF mass spectra of single-base extension (SBE) products from 20% *C. deserticola* in powder mixture of *C. deserticola* and *C. tubulosa* using reaction 1 and reaction 2.

**FIGURE S8 ** MALDI-TOF mass spectra of single-base extension (SBE) products from 10% *C. deserticola* in powder mixture of *C. deserticola* and *C. tubulosa* using reaction 1 and reaction 2.

**FIGURE S9 ** MALDI-TOF mass spectra of single-base extension (SBE) products from 1% *C. deserticola* in powder mixture of *C. deserticola* and *C. tubulosa* using reaction 1 and reaction 2.

**FIGURE S10 ** Representative pictures of dried specimens morphically resembling *Cistanche* species.
